# Occult Form of Premature Ovarian Insufficiency in Women with Infertility and Oligomenorrhea as Assessed by Poor Ovarian Response Criteria

**Published:** 2017

**Authors:** Rubina Izhar, Samia Husain, Suhaima Tahir, Sonia Husain

**Affiliations:** 1- Department of Gynaecology and Obstetrics, Abbasi Shaheed Hospital and Karachi Medical and Dental College, Karachi, Pakistan; 2- Aziz Medical Center, Karachi, Pakistan

**Keywords:** Female infertility, Female infertility, Occult premature ovarian insufficiency, Oligomenorrhea, Ovarian reserve, Premature ovarian ageing, Premature ovarian failure

## Abstract

**Background:**

The purpose of this study was to evaluate the ability of poor ovarian response criteria to classify women presenting with infertility and oligomenorrhea as having “occult” premature ovarian insufficiency.

**Methods:**

This was a cross sectional study conducted at Aziz Medical Center, Karachi, Pakistan from 1st August 2015 to 31st July 2016. Women with infertility and oligomenorrhea were included. All eligible women underwent day 2 FSH level and an early follicular phase transvaginal ultrasound to assess the antral follicular count (AFC). All women then underwent the confirmatory test, of Anti-Mullerian Hormone (AMH) level. The main outcome measure was assignment to occult premature ovarian insufficiency (POI) after screening that used the criteria set out in fertility guideline for predicting the likely ovarian response to gonadotrophin stimulation. Another measure was to compare the sensitivity and specificity of the two index criteria, of FSH and AFC, relative to the emerging reference standard, of the AMH criterion.

**Results:**

The three criteria together classified 59 (34.91%) women as occult POI in those with oligomenorrhea. The sensitivity, specificity, negative predictive value and positive predictive value of FSH relative to AMH for these women were 77.8%, 95.7%, 90.2% and 89.4%, respectively whereas the same values of AFC relative to AMH were 92.6%, 99.1 %, 96.6% and 98%, respectively.

**Conclusion:**

Women with menstrual irregularity and infertility are at a higher risk for satisfying criteria of poor ovarian response irrespective of age. A policy incorporating these surrogate markers can be used to screen these women for occult premature ovarian insufficiency.

## Introduction

Premature ovarian insufficiency (POI) previously known as premature menopause or premature ovarian failure (POF) is surprisingly not a rare occurrence (1). It affects 1 in 100 women of age greater than 40 years and 1 in 1000 women aged less than 30 years (2). European society of human reproduction and embryology (ESHRE) describes POI as the presence of amenorrhea for 4 months or more before the age of 40 in women, accompanied with a serum FSH level of >25 *IU*/l on two occasions four weeks apart (3).

The main presenting symptom of the condition is amenorrhea but due to residual function of ovarian follicles, around half of the women have oligomenorrhea and spontaneous ovulations (4). According to an estimate, 5–10% of women with POI conceive spontaneously. These women are said to have a variant of POI which is called “occult primary ovarian insufficiency” associated with diminished ovarian reserve (DOR) (5). Occult ovarian failure was first described as a triad of regular menses, infertility and high plasma levels of Follicle Stimulating Hormone (FSH) (6). These women with occult POI are not recognized until their presentation of infertility (5). Current literature on screening for occult premature ovarian insufficiency or diminished ovarian reserve (DOR) in these women is sparse. Studies that support screening to recognize early form of POI are limited to certain high risk populations (7). Moreover, there is no standard definition or criteria to diagnose DOR (8). The term that comes closest to defining DOR is poor ovarian response (POR) and can be used as a surrogate marker. The Bologna ESHRE consensus labels women as “poor ovarian responders” when at least two of the following three criteria are satisfied: (i) advanced maternal age (≥40 years) or any of the risk factors for POR, (ii) a previous poor ovarian response (≤3 oocytes with a conventional stimulation protocol), and (iii) an abnormal ovarian reserve test (*i.e*., antral follicular count (AFC) <5–7 follicles or Anti Mullerian Hormone (AMH) <0.5–1.1 *ng/ml*) (9). As a result, only women over 40 years of age or those who have previously had at least one controlled ovarian hyper stimulation cycle can be included. A young infertile woman with markers of poor ovarian reserve who has never undergone ART does not meet the ESHRE criteria. According to the National Institute of Health and Care Excellence (NICE) guideline on fertility, any one of the following (i) an FSH level of ≥8.9 *IU/L*, or (ii) AMH level of ≤5.4 *pmol/l* (≤0.7 *ng/ml*) or (iii) AFC of ≤4 predicts a likely low ovarian response to gonadotropin stimulation in an IVF cycle and is a predictor of diminished ovarian response (10). Because this prediction strategy does not include age or prior stimulation result, it can be chosen as standard for studying women at risk of premature ovarian insufficiency (POI).

A woman presenting with menstrual irregularity appears more at risk of having occult POI, as compared to one without any such pointers in history. We undertook this study to assess the hypothesis that these women would have poorer reserves and can benefit from screening.

The aim of this study was to evaluate the ability of poor ovarian response criteria set out in NICE guideline to classify women presenting with infertility and oligomenorrhea as having occult premature ovarian insufficiency.

## Methods

Women, aged 20 to 39 years with infertility and oligomenorrhea were recruited for this study from infertility clinic at Aziz Medical Centre in Karachi, Pakistan, from 1st August 2015 to 31st July 2016. Infertility was defined as the inability to conceive after 24 months of regular unprotected sexual intercourse where semen analysis was reported within normal limits. Women with oligomenorrhea which was defined for the study purpose as at least three menstrual cycle lengths of more than 40 days during the preceding year were selected as target population. Excluded from the study were those with history of cancer treatment, known autoimmune disorders, prior treatment of endometriosis or surgery to reproductive tract, prior history of pelvic inflammatory disease, those with tubal factors and uterine factors as assessed on history and confirmed by normal hysterosal-pingogram. Also, women who satisfied the Rotterdam’s criteria for polycystic ovarian syndrome (PCOS) were excluded from the study (11).

After obtaining written and informed consent, all women who met the inclusion criteria were scheduled for an infertility workup. In all women, pregnancy was excluded by negative serum beta-HCG levels (<1.2 *mU/ml*).

After that, an infertility workup panel consisting of day 2 FSH level and an early follicular phase transvaginal ultrasound to assess the antral follicular count (AFC) was performed. Transvaginal ultrasound on menstrual cycle days 4–8 was used to assess the antral follicle count and follicles of ≤5 *mm* as measured by transvaginal ultrasound were included in the count in all women.

All women then underwent the confirmatory test, of AMH level. For estimation of serum AMH levels, all selected subjects’ blood samples were drawn by venipuncture in serum separator tubes. Blood samples were taken for AMH levels on any day of the menstrual cycle. Serum AMH levels were determined by enzyme-linked immuno-sorbent assay (ELISA), using Human AMH Elisa kit (CDN-E 1350, Beckman Coulter, Chaska, MN, USA) at the reference lab.

Women were diagnosed with occult premature ovarian insufficiency if they had an FSH level of ≥8.9 or had AMH level of ≤5.4 *pmol/l* (≤0.7 *ng/ml*) or total ovarian antral follicle count of ≤4 as per NICE criteria (10).

A proforma was used to collect the data. The demographic data included age, height and weight of women, and area of residence. Past history regarding duration of infertility, age of menarche, type of infertility and duration of menstrual irregularities was also noted. The FSH level, antral follicle count (AFC) on initial scan and AMH level were also recorded in the performa.

The primary outcome measure in this study was to assign women with infertility and oligomenorrhea to occult POI after the initial screening using the criteria set out in fertility guideline for predicting the likely ovarian response to gonadotrophin stimulation in IVF by NICE. The secondary outcome measure was to compare the sensitivity and specificity of the two index criteria, FSH and AFC, relative to the emerging reference standard, the AMH criterion.

All participants provided informed consent. In lieu of formal ethics committee or formal institutional review board approval, Helsinki’s declaration was followed. No subjects were harmed, confidentiality was maintained and no subject was enrolled in the study without formal informed consent.

### Statistical analysis:

Data was entered and analyzed using SPSS version 15. Shapiro Wilk’s test was used to assess normality of data distribution. The quantitative variables including age, duration of infertility, duration of menstrual irregularities, FSH level on day 2 of cycle, antral follicular count on day 2, anti mullerian hormone (AMH) level, age of menarche in years, body mass index and difference between duration of menstrual irregularity and duration of infertility were presented by medians and range. Mann Whitney u test was used to compare groups. Frequency and percentages were computed for qualitative variables, type of infertility and area of residence. Chi square test and Fischer’s exact test were used to compare these variables at p<0.05 level of significance.

A 2×2 contingency table was used to assess sensitivity, specificity, positive predictive value and negative predictive value of both FSH level criteria and AFC level criteria relative to AMH criteria. AMH criterion was selected as a reference to compare the other two criteria because of growing evidence favoring its utility as the marker of choice for DOR (12).

The kappa statistic was used to ascertain agreement between the FSH level criteria and AMH level criteria, and the AFC criteria and AMH criteria.

SPSS version 15.0 (SPSS Inc., Chicago, IL, USA) was used for all statistical analysis.

## Results

During the study period, 169 women satisfied the inclusion criteria. The baseline characteristics of women are presented in table 1. The median age at presentation was 31 years (range 20 to 38 years). The median duration of infertility was 3 years (range 2 to 7 years). The median duration of menstrual irregularity in those was 5 years (range 2 to 9 years).The menstrual irregularity predated infertility by a median of 2 years.

**Table 1.** Characteristics of studied women and stratified according to reserves

**Characteristics**	**N=169**	**Normal ovarian reserve** **n=110**	**Diminished ovarian reserve** **n=59**	**P-value** ^†^
Age at presentation (years)	31(20–38)	31(20–38)	32(20–38)	0.342
Duration of infertility (years)	3(2–7)	3(2–7)	3(2–7)	0.774
Duration of menstrual irregularities (years)	5(2–9)	5(2–9)	4(2–9)	0.256
FSH level on day 2 of cycle (*IU/l*)	6(5–30)	6(5–7)	10(6–30)	<0.001^*^
Antral follicular count	5(0–7)	6(5–7)	2(0–6)	<0.001^*^
Anti mullerian hormone (AMH) level (*ng/ml*)	1.10(0.19–1.90)	1.20(0.80–1.90)	0.51(0.19–1.50)	<0.001^*^
Age of menarche (years)	13(11–15)	12(11–15)	13(11–15)	0.667
BMI (*kg/m*^2^)	22.65(19.08–29.28)	22.65(19.08–29.28)	22.65(19.08–29.28)	0.824
Difference between duration of menstrual irregularity and duration of infertility	2.00(−1.00–+4.00)	2.00(−1.00–+4.00)	1.00(−1.00–4.00)	0.125
Type of infertility (n, *%)*				0.162
Primary	141(83.4%)	95(86.4%)	46(78.0%)	
Secondary	28(16.6%)	15(13.6%)	13(22.0%)	
Area of residence (n, *%*)				0.871
Urban	127(75.1%)	83(75.5%)	44(74.6%)	
Suburban	23(13.6%)	14(12.7%)	9(15.3%)	
Rural	19(11.2%)	13(11.8%)	6(10.2%)	

Values are median range unless otherwise specified;

†: chi square test, Fischer’s exact test or Mann Whitney U test;

*: p-value is significant at less than 0.05

There was no statistically significant difference that could be seen in any characteristics between those who satisfied the criteria for occult premature ovarian insufficiency and those who did not satisfy any criteria (Table 1).

The FSH level on day 2 of cycle, AFC and AMH level were significantly lower in those with occult POI (p<0.001) (Table 1).

In the target population, 59 women satisfied at least one criterion that was set out to label diminished ovarian reserve. Thus, using the criteria set out to diagnose poor ovarian response, about 34.91% were put in the category of occult premature ovarian insufficiency. In the study group, 47 women satisfied the criterion set according to FSH level, whereas when antral follicles count criteria were used, 50 women were identified with diminished reserve. The AMH level criterion was satisfied by 54 women (Table 2).

**Table 2.** Percentage of women satisfying criteria

**Women with oligomennorhea (n=169)**	**n**	**%**
FSH ≥8.9	47	27.8%
AFC ≤4	50	29.6%
AMH ≤0.7	54	32.0%
FSH ≥8.9 OR AFC ≤4 OR AMH ≤0.7	59	34.91%
FSH ≥8.9 AND AFC ≤4	39	23.07%
FSH ≥8.9 OR AFC ≤4	58	34.3%
FSH ≥8.9 AND AMH ≤0.70	42	24.85%
FSH ≥8.9 OR AMH ≤0.70	59	34.91%
AFC ≤4 AND AMH ≤0.70	50	29.58%
AFC ≤4 OR AMH ≤0.70	54	32.0%
FSH ≥8.9 AND AFC ≤4 AND AMH ≤0.7	39	23.07%

The sensitivity, specificity, negative predictive value and positive predictive value of FSH relative to AMH for the target population were 77.8%, 95.7%, 90.2% and 89.4%, respectively (Table 3).

**Table 3.** AFC criteria * AMH criteria cross tabulation

**FSH**	**AMH**		**Total**

	**Yes**	**No**	
**Yes n(%)**			
	42 (77.8)	5 (4.3)	47 (27.8)
**No n(%)**			
	12 (22.2)	110 (95.7)	122 (72.2)
**Total n(%)**			
	54 (100)	115 (100)	169 (100)

The sensitivity, specificity, negative predictive value and positive predictive value of AFC relative to AMH for cases were 92.6%, 99.1%, 96.6% and 98%, respectively (Table 4).

**Table 4.** AFC criteria * AMH criteria cross tabulation

**AFC**	**AMH**		**Total**

	**Yes**	**No**	
**Yes n(%)**			
	50 (92.6)	1 (0.9)	51 (30.2)
**No n(%)**			
	4 (7.4)	114 (99.1)	118 (69.8)
**Total n(%)**			
	54 (100.0)	115 (100.0)	169 (100.0)

The kappa statistic of 0.76 was calculated between FSH level criterion and AMH level criterion as agreement on diagnosing occult premature ovarian insufficiency in women with oligomenorrhea and infertility while it was 0.93 between AFC and AMH for the same women.

## Discussion

The present study evaluated the utility of poor ovarian response to ovarian stimulation criteria by NICE for screening occult premature ovarian insufficiency in women presenting with infertility and oligomenorrhea. Our study showed that similar criteria can be used to screen women with occult variant of POI.

The criteria identified 59 (34.91%) women with occult POI in those with oligomenorrhea, the high risk group.

The AMH criterion was fulfilled by 54 (32%) women with oligomenorrhea followed by AFC criterion, 50(%), and FSH level criterion, 47 (%).

Relative to the AMH criteria, FSH criteria were less sensitive than AFC criteria for screening occult POI (77.8% versus 92.6%). The AFC criteria had excellent agreement with AMH criteria (kappa 0.93) whereas the FSH criteria had good agreement (kappa 0.76).

### Strength and limitations:

Our study is the first to assess the standard criteria recommended for screening poor response to ovarian stimulation by NICE as a surrogate marker for diagnosing women with possible occult POI.

All the three commonly used criteria were used to assess agreement between them.

The major limitation was that the ovaries were not simulated and the emerging gold standard, i.e. AMH, was used as the reference in its place for comparisons. In the absence of the specific definition and criteria to label a case as occult POI, and the likelihood that woman may still ovulate on her own, ready-made cut offs from the screening recommendations were used.

### Interpretation:

POI represents one extreme of the spectrum while the other end is represented by an asymptomatic woman with occasional menstrual irregularity (2). Women with an occult form of POI may have spontaneous follicular activity, and if hormonal tests are done during these times, levels of FSH and estradiol could be normal, or FSH could be only minimally raised. These investigations allow the occult variant to remain hidden, unless an analysis of ovarian reserve is carried out (5). These women have accelerated loss of ovarian follicles and have a narrower window of conceiving as compared to other women of the same age. Active case finding and aggressive course of treatment can enable these women to have their own children in time (13, 14). Thus, it is an entity that has an asymptomatic stage that can be recognized on ovarian reserve testing and lower the emotional and financial cost of the proposed treatment, the IVF later on, in short, it merits screening (15).

The menstrual irregularity starts prior to other features and may be subtle. In our study, the menstrual irregularity predated infertility by a median of 2 years in those presenting with oligomenorrhea. This is similar to a recent study from Russia, where the mean period of oligomenorrhea before diagnosis was 1.8 years in those presenting with infertility (16). This can be considered the first marker in the natural history of the disease and can narrow down the population that may benefit from ovarian reserve screening (17).

In the absence of stringent definition and universal criteria for diagnosing diminished reserve, screening these women was more difficult than anticipated. Utilizing surrogate criteria in our analysis, 34.91% women presenting with oligomenorrhea and infertility were screened to have diminished reserve, which is a bit higher than the reported prevalence worldwide of 10% to 30% (18).

Even this apparently high percentage is more likely to be an underestimate as the criteria used are derived from pooled results of studies evaluating poor response to ovarian stimulation in most older women. For women of similar age with accelerated loss of ovarian function, these values would have to be adjusted beyond their usual cut-off.

The ESHRE criteria for diagnosing POR use only AMH and AFC levels in addition to age and prior poor response (9). Our analysis also shows an inclination towards adoption of AFC and AMH levels as compared to FSH levels in these women. The FSH criterion was less sensitive than AFC (77.8% versus 92.6%) when compared against AMH. In their analysis on women with POI, Shestakova et al. also showed that FSH levels have no clinical significance and should not be used to assess the reserve in these selected cases (16, 19).

The AMH level is slowly emerging as the standard for diagnosing diminished ovarian reserve but has not yet received the label of gold standard (20, 21). The lack of consensus on the assay of choice for AMH and the cut-off for diagnosis is often quoted as a reason (22, 23). To overcome this, all the criteria endorsed by NICE were used and they were compared with AMH criterion. This enabled us to compare the AMH criterion with both, the most commonly used FSH criterion and the criterion that renders itself more suitable for adoption in those with just menstrual irregularities, *i.e*. the AFC criterion (24).

The AFC criterion showed excellent agreement with AMH criterion and does not suffer the assay of choice controversy (25). AFC criteria have been proved to fare better than all criteria according to some published literature (26, 27). Our study results are in agreement with this finding.

A likely plus point for adoption of the AFC criteria is that it can easily be incorporated into work-up for women presenting for menstrual irregularity as a part of the transvaginal scan. It can help to counsel them regarding their fertility status and serve the true purpose of a screening strategy (28).

Another point that merits discussion is that in women younger than 40 years, presenting with menstrual irregularity and infertility, the diagnosis of occult primary ovarian insufficiency was not uncommon. A screening strategy can help them decide sooner than later and allow an informed decision even in women presenting with the first tell-tale sign, the menstrual irregularity (29).

## Conclusion

A consensus for defining diminished ovarian reserve is the need of the hour so that these women can be screened efficiently. AMH is still an emerging standard and not the accepted standard. Our work shows that although FSH may still be within normal limits, the AMH is better suited to deal with these cases. Adoption of AMH and AFC criteria by NICE was proposed for screening women with infertility and menstrual irregularities and encouraging adoption of AFC for those presenting with menstrual irregularities was the best choice to generate data for screening policy.

Women with menstrual irregularity and infertility are at a higher risk for satisfying criteria of poor ovarian response irrespective of age. The same criteria except FSH level can be used as a surrogate for screening occult premature ovarian insufficiency in these selected cases. Age is not the sole marker of ovarian reserve in this population and screening can facilitate an informed decision regarding childbearing and outcome of fertility treatments in these women.

The response to ovarian stimulation in these patients needs to be assessed through further studies.
